# Homozygous mutation in *DNALI1* leads to asthenoteratozoospermia by affecting the inner dynein arms

**DOI:** 10.3389/fendo.2022.1058651

**Published:** 2023-01-16

**Authors:** Yanwei Sha, Wensheng Liu, Hua Nie, Lu Han, Chunjie Ma, Xiaoya Zhang, Ziyi Xiao, Weibing Qin, Xiaoming Jiang, Xiaoli Wei

**Affiliations:** ^1^ Department of Andrology, Women and Children’s Hospital, School of Medicine, Xiamen University, Xiamen, Fujian, China; ^2^ Fujian Provincial Key Laboratory of Reproductive Health Research, School of Medicine, Xiamen University, Xiamen, Fujian, China; ^3^ State Key Laboratory of Molecular Vaccinology and Molecular Diagnostics, School of Public Health, Xiamen University, Xiamen, Fujian, China; ^4^ National Health Commission (NHC) Key Laboratory of Male Reproduction and Genetics, Guangdong Provincial Reproductive Science Institute (Guangdong Provincial Fertility Hospital), Guangzhou, China; ^5^ School of Pharmaceutical Sciences, State Key Laboratory of Cellular Stress Biology, Xiamen University, Xiamen, Fujian, China; ^6^ School of Medicine, Yunnan University, Kunming, Yunnan, China; ^7^ Reproductive Medicine Center, Xiamen University Affiliated Chenggong Hospital, Xiamen, Fujian, China

**Keywords:** asthenoteratozoospermia, whole-exome sequencing, *DNALI1*, inner dynein arms, intracytoplasmic sperm injection

## Abstract

Asthenozoospermia is the most common cause of male infertility. Dynein protein arms play a crucial role in the motility of sperm flagella and defects in these proteins generally impair the axoneme structure and affect sperm flagella function. In this study, we performed whole exome sequencing for a cohort of 126 infertile patients with asthenozoospermia and identified homozygous *DNALI1* mutation in one patient from a consanguineous family. This identified homozygous mutation was verified by Sanger sequencing. *In silico* analysis showed that this homozygous mutation is very rare, highly pathogenic, and very conserved. Sperm routine analysis confirmed that the motility of the spermatozoa from the patient significantly decreased. Further sperm morphology analysis showed that the spermatozoa from the patient exhibited multiple flagella morphological defects and a specific loss in the inner dynein arms. Fortunately, the patient was able to have his child *via* intracytoplasmic sperm injection treatment. Our study is the first to demonstrate that homozygous *DNALI1* mutation may impair the integration of axoneme structure, affect sperm motility and cause asthenoteratozoospermia in human beings.

## Background

More than 80% of male infertility cases exhibit asthenozoospermia, which is caused by the dysfunction of sperm motility, such as reduced or completely absent sperm motility in the ejaculated (1, 2). Many factors, such as infection, varicocele, and pollution exposure, may predispose to asthenozoospermia. However, the genetic factors underlying asthenozoospermia cannot be ignored (3, 4).

The sperm flagellum plays an essential role in sperm motility through its conserved axonemal structure (5). Sperm axonemes consist of highly ordered “9+2” microtubules characterized by a central pair of microtubules surrounded by nine peripheral microtubule doublets (MTD) (6). There are various protein complexes, such as radial spokes, nexin-dynein regulatory complex, central complex, and dynein arms, as major components of the axoneme (7). Among these important complexes, dynein arms consisting of an inner and an outer dynein arm (IDA and ODA, respectively) are attached to each of the nine MTDs, which are essential for generating the beating forces of sperm flagellum (8). Strikingly, each dynein arm possesses a similar molecular composition: several light-chain proteins, at least two heavy-chain proteins, and at least two intermediate-chain proteins (9–11).

Dynein axonemal light intermediate chain 1 (*DNALI1*), also called *P28*, encodes a flagellar protein that is essential for the assembly of the inner dynein arm (12). Previous studies have shown that *p28* mutation disrupted dynein heavy chain composition in *Tetrahymena thermophila*, leading to defects in beat frequency and waveform patterns of cilia (13). Furthermore, DNALI1 is strongly expressed in spermatocytes, spermatids, and flagella of mature sperm in the murine testis, indicating its potential function in male reproduction (12). Based on structural analysis, DNALI1 was found to be linked to the C-terminus of DNAH1, and infertile patients with *DNAH1* mutations also presented DNALI1 defect in human beings (12, 14). Unfortunately, the role of DNALI1 in male reproduction has not been reported.

In the present study, we conducted whole-exome sequencing on 126 patients with asthenozoospermia and identified a homozygous mutation in *DNALI1* from an infertile patient. The spermatozoa of this patient showed motility and morphological defects, as well as a significant loss of the internal dynein arms. Our findings proposed that mutation in *DNALI1* is novel genetic pathogenesis of asthenoteratozoospermia and this infertile defect can be overcome by ICSI for the first time. These results demonstrate that DNALI1 plays an important role in the motility of sperm flagellum, which may extend the spectrum of etiological genes and provide new insight into the diagnosis and treatment of patients with asthenoteratozoospermia.

## Methods

### Subjects

We recruited 126 patients who were infertile due to asthenozoospermia for genetic analysis and 60 fertile healthy men as control subjects. The parents of the *DNALI1* mutated patient had consanguineous marriage. All infertile patients included in the study were excluded for abnormal karyotype, translocations, Y chromosome microdeletions, etc. We performed a routine analysis of semen for the participants.

### Ethical approval

This study was approved by the Ethics Committee of Women and Children’s Hospital of Xiamen University. All subjects participating in the study signed a written informed consent form.

### Whole-exome sequencing and Sanger sequencing

Whole-exome sequencing (WES) was performed on these asthenozoospermia patients as previously described (15). Briefly, genomic DNA for each patient was isolated from the peripheral blood sample and processed for exome enrichment using the TruSeq Exome Enrichment kit according to the manufacturer’s protocol. DNA sequencing was performed on an Illumina Hiseq 2000 sequencer and sequence reads were aligned to the human genome reference (hg19) using Burrows-Wheeler Aligner and sorted by Picard software. Candidate variants were annotated by using ANNOVAR and other bioinformatics databases. Further Sanger sequencing was performed to validate the selected mutation site in the *DNALI1* mutated patient. We were unable to validate this mutation site in his parents because both of his parents passed away.

### Transmission electron microscopy

Transmission electron microscopy (TEM) was performed at the core facility of biomedical sciences of Xiamen University as described elsewhere (16). Briefly, the fresh spermatozoa were first fixed by incubation in 2.5% glutaraldehyde. The samples were washed twice with 0.1M phosphate buffer and resuspended in 0.2 M sodium cacodylate buffer. After embedding with Epon 812, the ultrathin sections were stained with uranyl acetate and lead citrate and observed by TEM (JEM-1400, Jeol, Japan).

### Intracytoplasmic sperm injection

Intracytoplasmic sperm injection (ICSI) treatment for assisted fertilization was performed as described previously (17). The percentage of fertilization was evaluated by the presence of two polar bodies and two pronuclei. Then the embryos were individually cultured in Vitrolife G-SERIES culture media. Serum HCG levels were measured 14 days after embryo transfer and clinical pregnancy was confirmed by ultrasound performed 28 days after embryo transfer.

## Results

### Identification of homozygous *DNALI1* mutation in a patient with asthenoteratozoospermia

**Figure 1:**
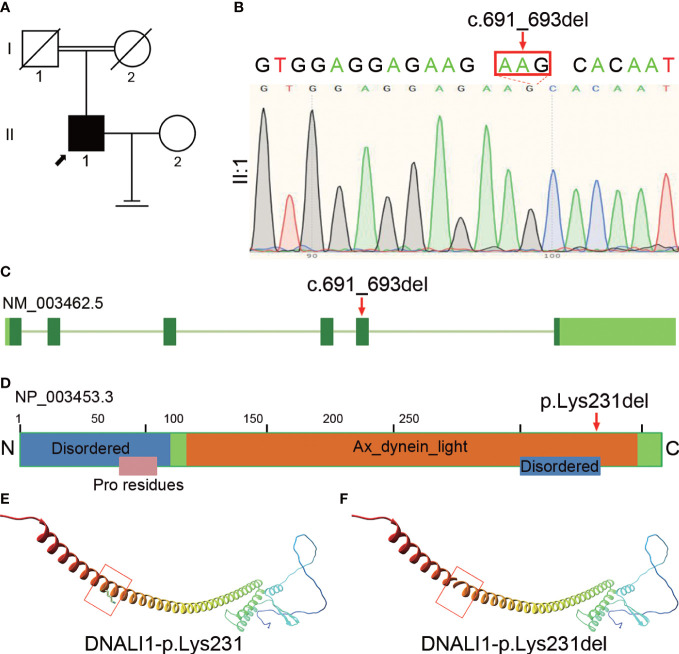
Identification of homozygous *DNALI1* mutation in an infertile man with asthenoteratozoospermia. **(A)** Pedigree chart of the patient with asthenoteratozoospermia. The black square and the black arrow represent the proband. **(B)** Sanger sequencing verified the variant in the patient. The mutated bases are indicated by the red arrow and red rectangle. **(C)** The location of the mutated bases on the genome of *DNALI1*. **(D)** The position of the amino acid substitution on the domain map of DNALI1. The blue rectangle represents the “Disordered” domain, the orange rectangle represents the “Ax_dynein_light” domain, and the pink rectangle represents the “Pro residues” domain. **(E)** The position of the affected amino acid on the three-dimensional structure of the wild-type DNALI1. The red rectangle shows p.Lys231 of DNALI1. **(F)** Effect of the deletion mutation on DNALI1 three-dimensional structure. The red rectangle shows the site of the deleted amino acid.

We performed WES and bioinformatic analyses to reveal the genetic etiology of 126 patients with asthenozoospermia and identified the homozygous *DNALI1* mutation NM_003462.5:c.691_693del in a patient from a consanguineous family **(** **Figure 1A** **)**. This homozygous *DNALI1* mutation in this patient was further confirmed by Sanger sequencing **(** **Figure 1B** **)**. Among all homozygous or compound heterozygous mutated genes in this patient, no genes that have been reported to be associated with asthenozoospermia, teratozoospermia, or structural components of spermatozoa were identified. Among the testis-specific or highly expressed genes, also only the DNALI1 gene was mutated and has been associated with primary ciliary dyskinesia. The ultrastructural defects of the inner dynein arms could be found. In *Chlamydomonas*, mutations in this gene exhibits similar defects. Moreover, by TEM, we also observed a defect in the inner dynein arms of axonemes in this patient. Based on these evidences, we hypothesize that *DNALI1* mutation is likely responsible for asthenoteratozoospermia in this patient.

This patient had some symptoms of suspected PCD such as cough, chronic sinusitis, and recurrent upper respiratory tract infections. Based on this, we hypothesized that this patient is a suspected PCD patient. This patient had a history of infertility for 5 years after marriage, but his parents had normal fertility with only one child. Although further verification was not available due to the passing of his parents, we hypothesized that the patient’s parents were heterozygous carriers of this mutation and that the patient’s homozygous mutation locus was likely inherited from his parents who had a consanguineous marriage.

### *In silico* analysis of the identified homozygous *DNALI1* mutation

The homozygous *DNALI1* mutation was further evaluated using *in silico* analysis. This *DNALI1* mutation NM_003462.5:c.691_693del (p.Lys231del) is very rare in the EXAC, 1000 genome, ESP6500, and gnomAD databases of the human population. This variant is classified as of uncertain significance with minor pathogenic evidence according to the ACMG Classification.

This homozygous NM_003462.5:c.691_693del (p.Lys231del) mutation is located at the fifth exon of the *DNALI1* genome **(** **Figure 1C** **)** and results in the deletion of the 231^th^ amino acid in the Ax_dynein_light and the Disordered domains **(** **Figure 1D** **)**. We then aligned the amino acid sequences of DNALI1 from *Homo sapiens* to that of *Drosophila melanogaster* and found that the amino acid affected by the NM_003462.5:c.691_693del (p.Lys231del) mutation is highly conserved among these species from human beings to fruit fly **(** **Supplementary Figure 1** **)**. In addition, we constructed the mutated protein structure using SWISS-MODEL and found that this mutation affected the three-dimensional structure of DNALI1. Compared to the original DNALI1 three-dimensional structure **(** **Figure 1E** **)**, the NM_003462.5:c.691_693del (p.Lys231del) mutation resulted in the deletion of Lysine at the 231^th^ **(** **Figure 1F** **).** Lysine is a positively charged basic hydrophilic essential amino acid of the basic amino acid class. This change could significantly affect the nearby steric hindrance and the three-dimensional structure of DNALI1, possibly affecting its stability and function.

### Spermatozoa defects of the patient with *DNALI1* deficiency

Clinical examination showed that the *DNALI1*-mutated patient had normal physical development. No significant abnormalities were found in the development of organs or accessory glands of the reproductive system. Serum hormone levels were within the normal range, with only a slight increase in PRL values **(** **Table 1** **)**. Routine semen analysis was performed for the patient, and the results showed that the patient had normal sperm concentration. However, the percentages of progressive motility and non-progressive motility were significantly decreased, and the percentage of normal morphology sperm was also lower than the reference value **(** **Table 2** **)**. We performed CASA on the patient’s spermatozoa, and the results showed that the patients had significantly lower sperm parameters **(** **Table 3** **)**.

**Table 1:** Clinical data of the patient harboring homozygous *DNALI1* mutation.

	Patient	Reference
Age (year)	32	–
Infertility (year)	5	
Height (cm)	166	–
Body weight (kg)	60	–
BMI	22.58	–
Testicular volume (Left/Right, ml)	12/12	10-15ml
FSH (mIU/ml)	10.18	1.27∼18.96
LH (mIU/ml)	8.23	1.24∼8.62
Testosterone (ng/ml)	4.51	4.14-7.26
PRL (ng/ml)	14.38	2.64∼13.13
E2 (pg/ml)	22	20∼75

**Table 2:** Semen parameters of the patient with asthenoteratozoospermia.

Patient	Volume(ml)	PH	Concentration(10^6^/ml)	Total sperm(10^6^)	PR (%)	NP (%)	PR+NP(%)	IM (%)	Normal forms (%)
First	3.0	7.20	16.42	49.25	15.67	0.75	16.42	83.58	3
Second	3.0	7.30	9.80	29.40	6.25	1.25	7.5	92.50	3
Reference	≥1.5	≥7.2	≥15	≥39	≥32	–	≥40	–	≥4

PR, progressive motility; NP, non-progressive motility; IM, immotility.

**Table 3:** Semen parameters of CASA of the patient with asthenoteratozoospermia.

Patient	VCL (μm/s)	VSL (μm/s)	VAP (μm/s)	ALH (μm)	LIN (%)	WOB (%)	STR (%)	BCF (Hz)	MAD (°)
First	26.98	22.62	23.13	1.80	83.28	85.56	96.87	5.25	35.85
Second	28.75	20.58	22.26	3.66	68.73	77.10	84.10	5.30	33.29

1. VCL, curvilinear velocity (m/s). The time-averaged velocity of a sperm head along its actual curvilinear path, as perceived in two dimensions in the microscope. A measure of cell vigor.

2. VSL, straight-line (rectilinear) velocity (m/s). The time-averaged velocity of a sperm head along the straight line between its first detected position and its last.

3. VAP, average path velocity (m/s). The time-averaged velocity of a sperm head along its average path. This path is computed by smoothing the curvilinear trajectory according to algorithms in the CASA instrument; these algorithms vary between instruments, so values may not be comparable among systems.

4. ALH, the amplitude of lateral head displacement (m). The magnitude of lateral displacement of a sperm head about its average path. It can be expressed as a maximum or an average of such displacements. Different CASA instruments compute ALH using different algorithms, so values may not be comparable among systems.

5. LIN, linearity. The linearity of a curvilinear path, VSL/VCL.

6. WOB, wobble. A measure of oscillation of the actual path about the average path, VAP/VCL.

7. STR, straightness. Linearity of the average path, VSL/VAP.

8. BCF, beat-cross frequency (Hz). The average rate at which the curvilinear path crosses the average path.

9. MAD, mean angular displacement (degrees). The time-averaged absolute values of the instantaneous turning angle of the sperm head along its curvilinear trajectory.

**Figure 2:**
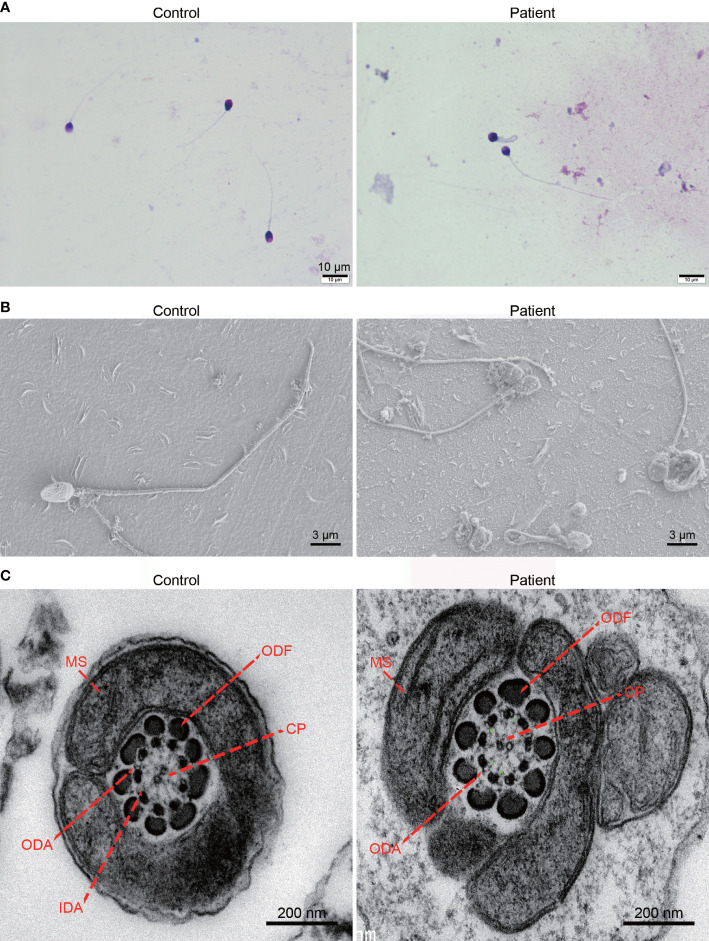
Morphological and ultrastructural analysis of the spermatozoa from the *DNALI1*-mutated patient. **(A)** Morphological analysis of the spermatozoa from a control subject and the patient with homozygous *DNALI1* mutation. Scale bar: 10 μm. **(B)** Morphological analysis of the patient’s spermatozoa by field emission scanning electron microscopy. Scale bar: 3 μm. **(C)** Ultrastructure analysis of the spermatozoa from the patient at the midpiece. The green “*” indicates the loss of IDA. Scale bar: 200 nm. CP, central pair of microtubules; ODF, outer dense fiber; MS, mitochondrial sheath; ODA, outer dynein arm; IDA, inner dynein arm.

Compared with the normal morphology of the spermatozoa from the control subject, the results of Papanicolaou staining showed that the patient’s spermatozoa exhibited multiple flagellar defects, including absent or coiled flagella **(** **Figure 2A** **).** The results of field emission scanning electron microscopy further confirmed the morphological defects of the patient’s spermatozoa **(** **Figure 2B** **).** In addition, TEM analysis was performed to explore ultrastructural defects, and it was found that the cross-section of the control sample showed a typical “9+2” axoneme structure, but the cross-sections of the patient’s spermatozoa exhibited specific inner dynein arm defects **(** **Figure 2C** **)**.

### Prognosis of the *DNALI1*-mutated patient following ICSI treatment

This couple underwent two cycles of ICSI treatment. In the first cycle, we retrieved 18 oocytes, 14 of which were in the MII stage. All these 14 oocytes at the MII stage were injected with the proband’s sperm and nine of them were fertilized. After embryo culture, only one blastocyst was formed on day 3. This embryo was transferred, but his wife failed to be conceived. In the second cycle, 14 oocytes were retrieved, nine of which were in the MII stage. All nine of these MII oocytes were injected with the proband’s sperm and all of them were fertilized. After embryo culture, two blastocysts were formed and transferred. The embryos were successfully implanted and his wife had a clinically successful pregnancy.

## Discussion

In this study, we recruited 126 infertile patients due to asthenozoospermia and detected a homozygous *DNALI1* mutation from one patient with asthenoteratozoospermia. This homozygous mutation in *DNALI1* resulted in the deletion of the inner dynein arms, which severely impairs sperm motility. These data suggest that *DNALI1* is a novel gene associated with sperm flagellar function and defects in this gene may contribute to asthenoteratozoospermia in humans.

The formation and function of sperm flagellum are essential for sperm motility (18). Typically, the sperm flagellum has a highly organized axoneme portion consisting of nine outer doublet microtubules and a central pair microtubule, called the “9+2” structure (19). Axonemal dyneins, including ODA and IDA, are observed on outer doublet microtubules, which play a central role in the beating and motility of sperm flagellum (20). Indeed, mutations in genes associated with the formation of the sperm tail are responsible for sperm motility and fertility defect (18). For example, mutations in IDA and ODA genes, such as *DNAH1*, *DNAH2*, *DNAH8*, and *DNAH10*, cause male infertility due to asthenozoospermia with multiple morphological abnormalities of the sperm flagella (21). However, mutations in *DNAH5*, *DNAH11*, and *DNAI1* also lead to male infertility due to isolated non-syndromic asthenozoospermia (22, 23). In this study, we identified for the first time homozygous *DNALI1* mutation as potential pathogenesis for asthenoteratozoospermia.

DNALI1 is a kind of axonemal IDA protein and is mainly expressed in the human ciliated tissues, including the testis, ovary, and lung (12). *DNALI1* mutation altered dynein heavy chain composition, which further resulted in defects in beat frequency and waveform patterns of cilia in *Tetrahymena thermophila* (13). In the present study, spermatozoa from the *DNALI1*-mutated patient exhibited specific inner dynein arm loss, resulting in a significant decrease in progressive and non-progressive motility. Moreover, CASA results further demonstrated that the motility parameters of the patient’s sperm were significantly reduced. In addition to the motility of spermatozoa, the flagella morphology of the patient’s sperm also exhibited various abnormalities characterized by absent or coiled flagella. These findings suggest that defects in *DNALI1* may affect IDA assembly during flagellar axoneme formation, leading to IDA deficiency, sperm flagellar morphology anomalous, and asthenozoospermia.

ICSI is the preferred clinical treatment for patients with asthenoteratozoospermia (24). However, for some patients with idiopathic asthenoteratozoospermia, multiple attempts are often required and some do not end up with satisfactory results (25). Therefore, reports on assisted reproduction are of clinical importance when studying infertility due to genetic defects. In this study, the patient underwent two cycles of ICSI treatment and obtained a clinical pregnancy, which could provide a reference for other infertility due to *DNALI1* deficiency.

## Conclusions

In summary, our work demonstrates that genetic defects of *DNALI1* severely impair sperm motility and contribute to the human azoospermia phenotype, for which ICSI treatment is an effective remedy for the first time. Our results prove the importance of DNALI1 in the structure and function of the sperm axoneme, which provides novel evidence for a comprehensive understanding of the axonemal assembly and function of sperm flagellum.

## Data availability statement

The datasets presented in this article are not readily available because the CNGB regulations. Requests to access the datasets should be directed to Y-WS, shayanwei928@126.com.

## Ethics statement

The studies involving human participants were reviewed and approved by the Ethics Committee of Women and Children’s Hospital of Xiamen University. The patients/participants provided their written informed consent to participate in this study.

## Author contributions

XW and XJ designed this study. XW and WL drafted the manuscript. YS, WQ, and HN performed bioinformatic analysis. XZ and ZX performed molecular genetics experiments. YS and XJ conducted clinical phenotyping. LH and CM interpreted the data. All authors approved the final manuscript.

## Funding

This work was supported by the following grants: the National Natural Science Foundation of China (82071697, and 81871200), the Medical Innovation Project of Fujian Province (2020-CXB-051), the open project of NHC Key Laboratory of Male Reproduction and Genetics in Guangzhou (KF202004).

## Acknowledgments

We express our deepest gratitude to the participants for their cooperation.

## Conflict of interest

The authors declare that the research was conducted in the absence of any commercial or financial relationships that could be construed as a potential conflict of interest.

## Publisher’s note

All claims expressed in this article are solely those of the authors and do not necessarily represent those of their affiliated organizations, or those of the publisher, the editors and the reviewers. Any product that may be evaluated in this article, or claim that may be made by its manufacturer, is not guaranteed or endorsed by the publisher.
