# Supplementary figures and images for: Homozygous mutation in DNALI1 leads to asthenoteratozoospermia by affecting the inner dynein arms
Source: Front Endocrinol (Lausanne). 2023 Jan 16;13:1058651. doi: 10.3389/fendo.2022.1058651 (PMC9885801; doi:10.3389/fendo.2022.1058651)

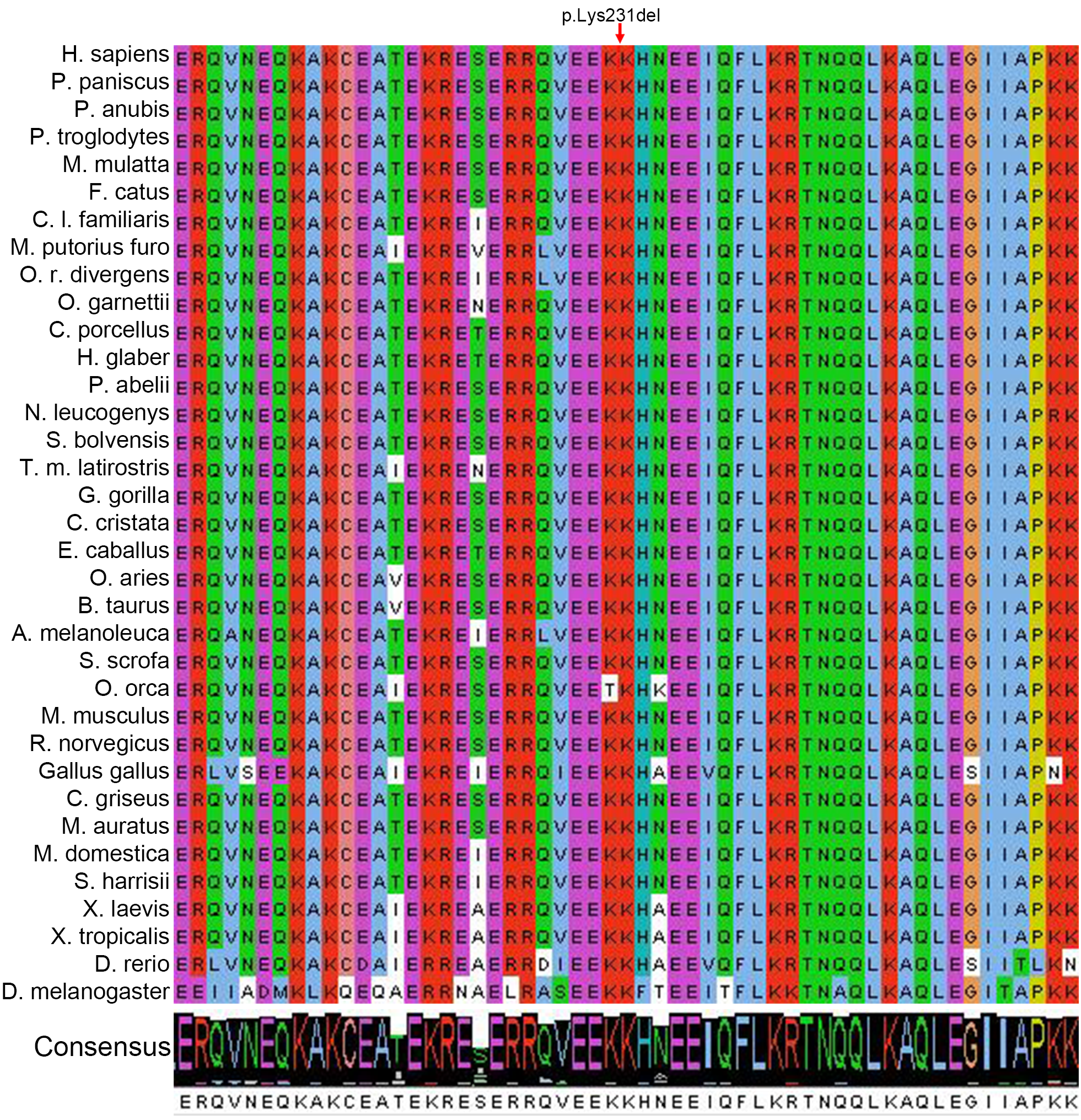

Supplement: Supplementary file 1 [file Image_1.tif]
